# Reactivation of Denervated Schwann Cells by Embryonic Spinal Cord Neurons to Promote Axon Regeneration and Remyelination

**DOI:** 10.1155/2019/7378594

**Published:** 2019-12-02

**Authors:** Xinyu Fang, Chaofan Zhang, Chongjing Zhang, Yuanqing Cai, Zibo Yu, Zida Huang, Wenbo Li, Wenming Zhang

**Affiliations:** Department of Orthopedic Surgery, First Affiliated Hospital of Fujian Medical University, Fuzhou, Fujian, China

## Abstract

In peripheral nerve injuries (PNIs) in which proximal axons do not regenerate quickly enough, significant chronic degeneration of Schwann cells (SCs) can occur at the distal stump of the injured nerve and obstruct regeneration. Cell transplantation can delay the degeneration of SCs, but transplanted cells fail to generate voluntary electrical impulses without downstream signal stimulation from the central nervous system. In this study, we combined cell transplantation and nerve transfer strategies to investigate whether the transplantation of embryonic spinal cord cells could benefit the microenvironment of the distal stump of the injured nerve. The experiment consisted of two stages. In the first-stage surgery, common peroneal nerves were transected, and embryonic day 14 (E14) cells or cell culture medium was transplanted into the distal stump of the CPs. Six months after the first-stage surgery, the transplanted cells were removed, and the nerve segment distal to the transplanted site was used to bridge freshly cut tibial nerves to detect whether the cell-treated graft promoted axon growth. The phenotypic changes and the neurotrophic factor expression pattern of SCs distal to the transplanted site were detected at several time points after cell transplantation and excision. The results showed that at different times after transplantation, the cells could survive and generate neurons. Thus, the neurons play the role of proximal axons to prevent chronic degeneration and fibrosis of SCs. After excision of the transplanted cells, the SCs returned to their dedifferentiated phenotype and upregulated growth-associated gene expression. The ability of SCs to be activated again allowed a favorable microenvironment to be created and enhanced the regeneration and remyelination of proximal axons. Muscle reinnervation was also elevated. This transplantation strategy could provide a treatment option for complex neurological injuries in the clinic.

## 1. Introduction

There are many serious traumas in the clinic that often lead to long segmental defects of the peripheral nerves without direct tension-free anastomosis repair methods. Autografts, acellular nerves, and allografts (ANAs) are alternative options [1, 2]. There are also cases limited by soft tissue conditions that require delayed repair [3]. However, these cases often have poor outcomes because the slow-growing proximal axons do not reach the distal nerve fast enough, and chronic degeneration of distal nerves and muscles hinders the potential for reinnervation [4, 5].

Currently, the most widely accepted clinical option to alleviate chronic degeneration is transferring an adjacent nerve to protect the distal nerve and muscles, known as the “babysit” procedure [6–9]. When the temporary neuroanastomosis is terminated months later and the original proximal end of the injured nerve stump (or including graft) is sutured back, the regenerated axons within can regrow into the distal stump [10]. However, these methods are characterized by the disadvantage of causing additional injury to the donor nerve, and sometimes, the source of the donor nerve is insufficient. Cell transplantation (including neural stem cells, embryonic spinal neurons, or in vitro-induced motor neurons) to the distal stump of peripheral nerve damage has been shown to delay chronic degeneration of the distal nerve and muscle [11]. However, transplanted cells fail to generate voluntary electrical impulses without downstream signal stimulation from the central nervous system [12]. In a previous study, we combined the “cell transplantation” and “nerve transfer” strategies to address this problem. We transplanted E14 neurons to the distal stump of transected nerves and showed that, 3 months after transplantation, after resection of the transplanted site, the ability of the distal nerve and muscle to support proximal axon regeneration was enhanced compared with the control group [13]. However, it remains unclear whether this improvement was induced by effects on the distal nerves, muscles, or both.

After peripheral nerve injury, Schwann cells (SCs) at the distal stump dedifferentiate and secrete growth-associated neurotrophic factors to induce proximal axonal regeneration [14]. However, this state is only temporary. If the regenerative axons are unable to contact the distal SCs within a certain time window, the SCs will enter a quiescent state, reducing the secretion of neurotrophic factors. Even if the proximal axon is in contact during this period, the distal SCs are unable to support elongation and remyelination. Sulaiman and Gordon confirmed that this time window was 6 months in rats [15]. In contrast, recent research believed that even if a rat has been denervated for more than 1 year, the muscle still has the ability to regenerate, which corresponds to better tolerance than nerves [16, 17]. Therefore, this study is aimed at determining the effect of transplanted embryonic neurons on SCs in distal nerve stumps in a rat peripheral nerve injury (PNI) model. We hypothesized that transplanted cells interact with SCs to maintain the microenvironment of the distal nerve stump in an axon-innervated condition. When these cells are removed, the nerves distal to the cells undergo another process that is equivalent to the denervation process, and the expression of GAGs rises again, creating a better environment for proximal axon regeneration. To verify this hypothesis, we designed the following experiment.

Briefly, the procedure consisted of two stages. In the first-stage (1^st^) surgery, the right common peroneal nerves (CPs) were transected, and embryonic day 14 (E14) cells or cell culture medium was transplanted into the distal stump of the CPs. The phenotype changes and neurotrophic factor expression of SCs distal to the transplanted site were measured at multiple time points after transplantation. Six months after the 1^st^ surgery, the transplanted cells were removed (second-stage surgery-I), and the phenotypic changes and neurotrophic factor expression pattern of SCs distal to the transplanted site were compared with the state after the 1^st^ surgery. Then, the nerve segment distal to the transplanted site was used to bridge the freshly cut tibial nerves (TIBs) to detect whether the nerve segment has the effect of promoting axon growth (second-stage surgery-II).

## 2. Materials and Methods

All surgical interventions and subsequent care and treatment were approved by the Ethics Committee for the Use of Animals at Fujian Medical University (No. SYXK-min-2016-0006). Animals were provided by the Laboratory Animal Unit at Fujian Medical University. We followed the methodology of our previously published work [13, 18, 19].

### 2.1. Cell Preparation

The Sprague-Dawley transgenic rats expressing green fluorescent protein (GFP; “green rat” CZ-004; SLC, Shizuoka, Japan) were used. The ventral spinal cord cells from embryonic day 14 (E14) rats were prepared with reference to a modified published protocol [20]. Briefly, under sterile conditions, the spinal cord was separated and the surrounding tissues were removed. The ventral horn of cord was collected and transferred to DMEM-F12 culture medium (Gibco, Grand Island, NY, USA). Trypsin (0.15%, Invitrogen, 25200-072, USA) was supplemented to the medium and digested for 7 minutes and then neutralized with trypsin inhibitor (Invitrogen, 17075-029, USA). Next, the spinal cord was dissociated into single-cell suspension by mechanical trituration and further filtered through a 40 *μ*m cell strainer (BD Falcon, San Jose, CA, USA). After centrifugation, the suspension was resuspended in DMEM-F12 culture medium at a density of 1.0 × 10^5^ cells/*μ*l and kept on ice for transplantation.

### 2.2. Staging Animal Surgery

**Figure 1:**
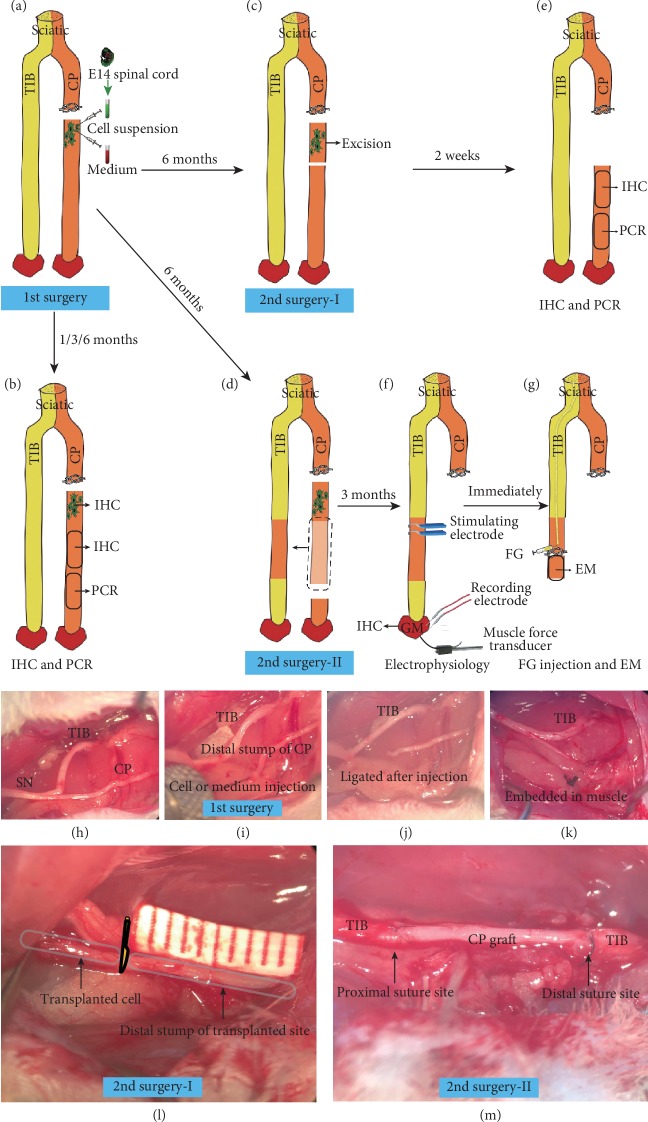
Schematic diagram of the stages of the surgery procedures. (a) In the first-stage surgery, the right CP was transected, and the proximal stump was ligated. Embryonic (E14) ventral spinal cord cells or an equivalent volume of cell culture medium was injected into the distal stump of the CP. (b) At different times (1, 3, or 6 months) after transplantation, IHC and PCR were performed using the nerve segments marked in the figure. (c) Six months after transplantation, the transplanted site was excised. (e) Two weeks later, the marked sites were processed for IHC and PCR. (d) Six months after transplantation, the transplanted site was excised, the marked segment was cut from the distal stump of the transplanted site, and then, the TIB was transected. The distal and proximal stumps of the TIB were bridged by this segment. (f) Three months after bridging surgery, electrophysiological examination was performed, and the GM was dissected for further IHC. (g) Immediately after electrophysiology, FG retrograde axonal tracing and EM analyses were performed. TIB: tibial nerve; GM: gastrocnemius muscle; IHC: immunohistochemical staining; EM: electron microscope; CP: common peroneal nerve; FG: fluorogold; EM: electron microscope.

Sixty female Sprague-Dawley rats (250-300 g) were used in this study. Animals were anesthetized with an intraperitoneal injection of ketamine (80 mg/kg) and xylazine (8 mg/kg) and placed in prone position. The surgeries were performed in two stages (Figure 1). In the first-stage (1^st^) surgery (nerve denervation and cell/vehicle injection), the animals were randomly divided into 2 groups. (i) Cell injection group: the CP was transected at a distance of 5 mm from the bifurcation of the sciatic nerve, and 3 *μ*l of cell suspension was slowly injected into the distal stump of the CP. The transplanted cells were confined to a limited area of the distal nerve to facilitate the second-stage resection of the transplanted area, and the proximal and distal stumps were ligated and imbedded into the muscle to prevent regeneration (Figures 1(a), 1(h), 1(i), 1(j), and 1(k)). (ii) Vehicle injection group: the same surgical procedure was performed, and 3 *μ*l of cell culture medium was injected (Figures 1(a), 1(h), 1(i), 1(j), and 1(k)). At different time points after the first surgery (1 month, 3 months, and 6 months), one-sixth of the rats in each group (*n* = 5) were killed, and 20 mm distal nerve segments including the transplanted site were collected for immunohistochemistry staining (IHC) and quantitative reverse transcription-PCR (qRT-PCR) analysis (Figure 1(b)).

Six months after the 1st surgery, the remaining rats underwent the second-stage (2nd) surgery (I-cell excision or II-cell excision plus distal nerve grafting). For the cell excision rats (*n* = 5), a 5 mm nerve segment including the transplanted cells was excised (2nd surgery-I, Figures 1(c) and 1(l)). The rats were maintained for an additional 2 weeks for the qRT-PCR analysis and IHC (Figure 1(e)). For the cell excision plus distal nerve grafting rats (*n* = 10), after the cells were excised, a 10 mm segment was cut from the distal stump of the transplanted site, and then, the TIB was transected. The distal and proximal stumps of the TIB were bridged by this segment with 10-0 sutures (2nd surgery-II, Figures 1(d) and 1(m)). The animals were kept for an additional 3 months, after which electrophysiology, fluorogold (FG) retrograde axonal tracing, and electron microscopy (EM) analyses were performed. The rats were maintained for an additional 3 days in favor of FG transportation (Figure 1(f)). Nerve segments of the same length were cut in the vehicle group (*n* = 10) during the second-stage surgery, and the subsequent procedures remained the same.

### 2.3. Immunohistochemistry Staining (IHC)

At every detection time point after the 1^st^ and 2^nd^ surgery-I, rats were euthanized and perfused with 4% paraformaldehyde (PFA). A 5 mm segment of CP containing the transplanted cells and a 10 mm adjacent segment were cut and collected (Figures 1(b) and 1(e)). The nerves were postfixed in 4% PFA at 4°C overnight and then dehydrated in 30% sucrose (dissolved in 0.1 M PB) at 4°C for 48 hours. Serial longitudinal cryosections (20 *μ*m thick) of the nerve segments were prepared. Every third section was used for IHC with reference to the published protocol [20] (5 rats per group per time point). For segments containing cells, staining of neuronal nuclei (NeuN) was performed. For adjacent distal segments, staining of neurofilament 200 (NF200, axon marker), myelin basic protein (MBP, myelin sheath marker), S100 (cytoplasmic marker of SCs), and P75 (dedifferentiated SCs marker) was performed. Briefly, after washed with 0.01 M phosphate-buffered saline (PBS), sections were blocked in 10% goat serum for 30 min and incubated with the primary antibodies at 4°C overnight (mouse anti-NeuN, 1 : 500, MAB377, EMD Millipore; mouse anti-NF200, 1 : 500, N0142, Sigma; rabbit anti-MBP, 1 : 200, M3821, Sigma; rabbit anti-S100, 1 : 200, Z0311, DAKO; rabbit anti-P75, 1 : 200, G3231, PROMEGA). After washed with 0.01 M PBS, sections were incubated with species-specific fluorescence-conjugated secondary antibodies at 20°C for 2 h (goat anti-mouse Alexa Fluor 586/488, 1 : 500, A11031/A11029, Invitrogen; donkey anti-rabbit Alexa Fluor 586/488, 1 : 500, A10042/A21206, Invitrogen). Sections were subsequently counterstained with 4′,6-diamidino-2-phenylindole (DAPI) to stain the nuclei and then coverslipped with antifade mounting medium (FluorSave, Calbiochem, San Diego, CA, USA).

The number of NeuN-positive cells was counted manually following a systematic random sampling technique [21]. Briefly, for all the measurements, one in 3 sections with a total of 6 sections was assessed. The counting was carried out in GFP-positive fields at ×40 magnification under a fluorescence microscope (Axioplan, Carl Zeiss, Germany). Cells that met all of the following criteria were counted: (1) cells that were present in the GFP-positive region and stained with NeuN and (2) cells that had a large cytoplasm and nucleus and contained at least one large nucleolus. The result is multiplied by 3 to yield the final count. The fluorescence intensity of NF200, MBP, S100, and P75 was evaluated in ImageJ software (National Institutes of Health, Bethesda, Maryland), which was presented as the mean gray values of the regions of interest (ROIs). In each sample evaluated, the mean gray value of ROI was normalized to the intensity of the uninjured CP and averaged over triplicate measurements from 1 rat for each time point [9].

At 3 months after the 2nd surgery-II, after the electrophysiological examination has been performed, the gastrocnemius muscle (GM) was dissected. The wet weight of GM was measured. And then the serial longitudinal cryostat sections (20 *μ*m) were prepared using a freezing microtome (Leica CM 1900). The sections were blocked in 5% normal donkey serum for 1 hour and incubated with primary antibodies at 4°C overnight (rabbit anti-neurofilament 200, 1 : 500, N4142, Sigma-Aldrich), followed by secondary antibodies at 37°C (Alexa Fluor 594, 1 : 500, A30008, Invitrogen). Finally, alpha-bungarotoxin conjugated to Alexa Fluor 488 (a-BTX, 1 : 500, B13422, Invitrogen) was applied to label acetylcholine receptors (AchRs). Every fifth section of the GM was used to count the neuromuscular junctions (NMJs) (10 sections per animal). The AchR clusters were captured, and 100 randomly selected NMJs were studied in each animal (10 animals in each group). The NMJs were categorized into reinnervated and denervated based on previously described methods with modifications [22]. The reinnervation rate was calculated as the number of reinnervated NMJs divided by the total number of NMJs.

### 2.4. Quantitative Real Time-PCR (qRT-PCR)

At every detection time point after the 1^st^ and 2^nd^ surgery-I, the expression levels of growth-associated genes (GAGs) in distal nerve, which has been previously shown to be significantly differentially expressed after denervation in vivo, were analyzed by qRT-PCR, including glial cell-derived neurotrophic factor (GDNF), brain-derived neurotrophic factor (BDNF), and nerve growth factor (NGF) [14]. The myelin-related gene myelin protein zero (MPZ) was also analyzed [23]. Additional contralateral intact CPs were used for normalization of the mRNA expression levels (i.e., day 0). Total RNA was extracted from the 5 mm CP segment using TRIzol reagent (Invitrogen, USA) following the manufacturer's protocol. The extracted RNA was further purified using DNase-I to eliminate residual genomic DNA. A total of 1 g of total RNA was reverse-transcribed using random primers and reverse transcriptase (M-MLV-RT, Takara, Japan) following the manufacturer's instructions. qRT-PCR was performed according to standard protocols using SYBR Green Kit (Takara, Japan) in the iCycler iQTM (Bio-Rad) system. Briefly, 1 *μ*l of cDNA was added to a 19 *μ*l reaction mixture containing 0.5 *μ*M primer sets and 0.5X SYBR Green mixture. The primers were designed and synthesized by Integrated DNA Technologies (Shatin, N. T., Hong Kong, China): NGF: forward 5′ACCTCTTCG-GACACTCTGGA3′ and reverse 5′GTCCGTGGCTGTGGTCTTAT3′; BDNF: forward 5′GAACAGGACGGAAACAGAACG3′ and reverse 5′GAACAGGACGGAAACAGAACG3′; GDNF: forward 5′GCGGTTCCTGTGAAGCGGCCGA3′ and reverse 5′TAGATACATCCACACCGTTTAGCGG3′; MPZ: forward 5′GGTGGTGCTGTTGCTGCTG3′ and reverse 5′TTGGTGCTTCGGCTGTGGTC3′; and *β*-actin: forward 5′-TCATGAAGTGTGACGTGGACATC-3′ and reverse 5′TGTTGCATTTGCGGG GACGATG-3′.

The PCR was run in the following cycling conditions: 95°C for 5 min and 35 cycles of 95°C for 30 s, 60°C for 30 s, and 72°C for 40 s. The expression level of each growth factor gene in the distal nerve was calculated as the fold change compared with that in the contralateral intact nerve (normalized to 1 arbitrary unit) using the 2-*ΔΔ*Ct method, where *Δ*Ct represents the difference between the Ct values of each gene and *β*-actin and *ΔΔ*Ct represents the difference in *Δ*Ct between the transected nerve and the intact nerve after normalization to *β*-actin. All PCRs were performed in triplicate and repeated for 3 times.

### 2.5. Electrophysiological Analysis

Three months after the 2^nd^ surgery-II, the functional recovery of the GM was evaluated by electrophysiological analysis via a standard nerve-evoked potential recording system (RM6240BD, Chengdu, China) in 10 animals per group. The procedure was performed as previously described and illustrated in Figure 1(f) [11, 13]. In brief, animals were anesthetized and placed in a prone position. The resutured TIB nerve and the right GM muscle were exposed. The bipolar stimulating electrode was placed underneath the nerve and kept contacted. The GM tendon was fixed to the transducer with a 4-0 suture to record muscle contraction force. For compound muscle action potential (CMAP) recording, the stainless-steel monopolar recording electrodes were inserted into the muscle belly, and the grounding electrode was inserted into the subcutaneous tissue. Initial electrical stimulus (0.1 mA; 1 ms duration; 1 Hz frequency; square wave) was applied and gradually increased by 0.1 mA until a supramaximal response was reached. The maximum muscle contraction force and the evoked CMAP were recorded for 5 times with a 2-minute interval. The same procedure was repeated on the contralateral side (L) which was set as the reference value. The results were presented as the ratio of the value on the surgical side (R) to that on the contralateral side (L) (R/L). After electrophysiological analysis, the GM muscle and distal nerve was collected. Subsequent retrograde axonal tracing was performed.

### 2.6. Electron Microscopy (EM) Analysis

At 3 months after the 2^nd^ surgery-II, after electrophysiology analysis, a 2 mm nerve segment from the distal part of the nerve graft was excised (10 animals per group) (Figure 1(f)). Nerves were postfixed in EM fixative (2.5% glutaraldehyde and 2% PFA in 0.1 M PB, pH 7.4) and 2% osmic acid and then dehydrated in gradient alcohol (30%–95%). Next, nerves were infiltrated and embedded in pure Epon at 60°C for 3 days. Semithin sections (1 *μ*m) were cut with glass knife in microtome and were then stained with 0.5% toluidine blue.

Sections were seen under microscopy and photos were taken. The number of myelinated axons was calculated using ImageJ. A total of 6 fields (0.5∗0.5 mm^2^) from each animal (10 animals per group) were randomly selected. The G-ratio, which was calculated by dividing the inner diameter of the axon (without myelin) by the outer diameter of the entire fiber (including the myelin), was calculated to evaluate the myelination of regenerating axons. At least 100 myelinated axons were randomly selected in each animal.

### 2.7. Retrograde Axonal Tracing and FG Counting

At 3 months after the 2^nd^ surgery-II, the regeneration of spinal cord motor neurons into the graft was examined by retrograde axonal tracing with FG (10 animals per group) [24]. In short, after the segment was excised for EM analysis (Figure 1(f)), 0.5 *μ*l of 2% (*w*/*v*) FG solution was injected proximally. The injection was slow and lasted for 10 seconds, after which the injection site was clamped with microforceps for 10 seconds and sutured to prevent leakage. The rats were sacrificed 3 days later to examine FG transportation. The L3-6 spinal cord was harvested, used, and processed for frozen section. Longitudinal sections (30 *μ*m thick) were evaluated under a fluorescence microscope (Axioplan, Carl Zeiss, Germany), and the FG-labeled neurons were counted. Two examiners independently counted the neurons with reference to the published protocol [24].

### 2.8. Statistical Analysis

Imaging analyses were evaluated in a blinded manner. Sections were randomly assigned identification numbers, and two experienced investigators (X Fang and C Zhang), respectively, assessed the slides. The values were averaged before further analysis. All the data are presented as the mean ± SEM. Two-way ANOVA followed by a post hoc Dunnett's test for multiple comparisons was carried out in the SPSS 13.0 software (Chicago, IL). A *P* value less than 0.05 was considered significant.

## 3. Results

### 3.1. Transplanted Neurons Survived in the Distal Stump of CPs at Different Time Points after Injection

**Figure 2:**
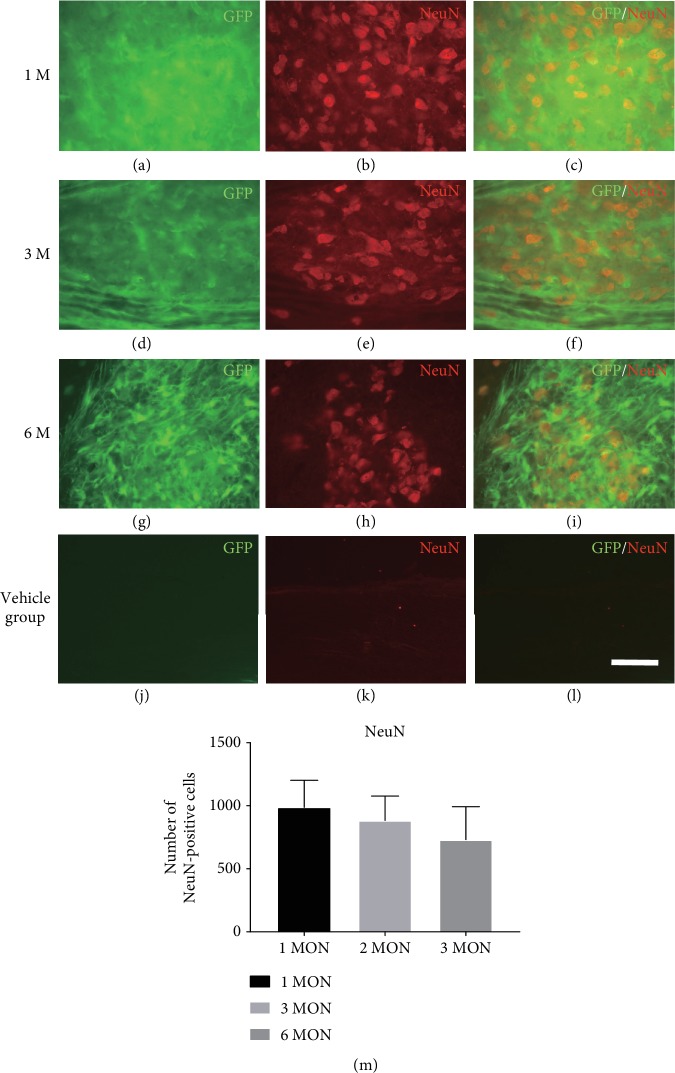
Neuronal survival at different time points after transplantation. A longitudinal section of the transplanted site with NeuN staining showed that many GFP-positive transplanted embryonic cells differentiated into NeuN-positive neurons at different time points. (a–c) One month after transplantation; (d–f) three months after transplantation; (g–i) six months after transplantation. (m) Comparing the mean numbers of NeuN-positive cells per time point revealed no significant differences. Scale bar = 50 *μ*m.

At different detection time points after the 1^st^ surgery, IHC was performed to confirm that the transplanted cells survived and differentiated to neurons at the injection sites. One month, three months, and six months after transplantation, many GFP-positive transplanted cells were observed at the distal stump of CPs (Figures 2(a), 2(d), and 2(g)). NeuN-positive neurons were abundant in the transplanted area (Figures 2(b), 2(e), and 2(h)). Cell counts revealed that the number of NeuN-positive cells gradually decreased over time, but there was no significant difference in the number of neurons among the three time points (*P* > 0.05). In the vehicle group, no GFP-positive area was found, and there were no NeuN-positive cells (Figures 2(j)–2(l)). Interestingly, we also noted that the intensity of GFP in NeuN-positive cells was somehow much weaker than that in NeuN-negative cells, similar to a previous report [20].

### 3.2. The Presence and Absence of Neurons Induced a Transition between the Denervated and Innervated States in the Distal Nerve

**Figure 3:**
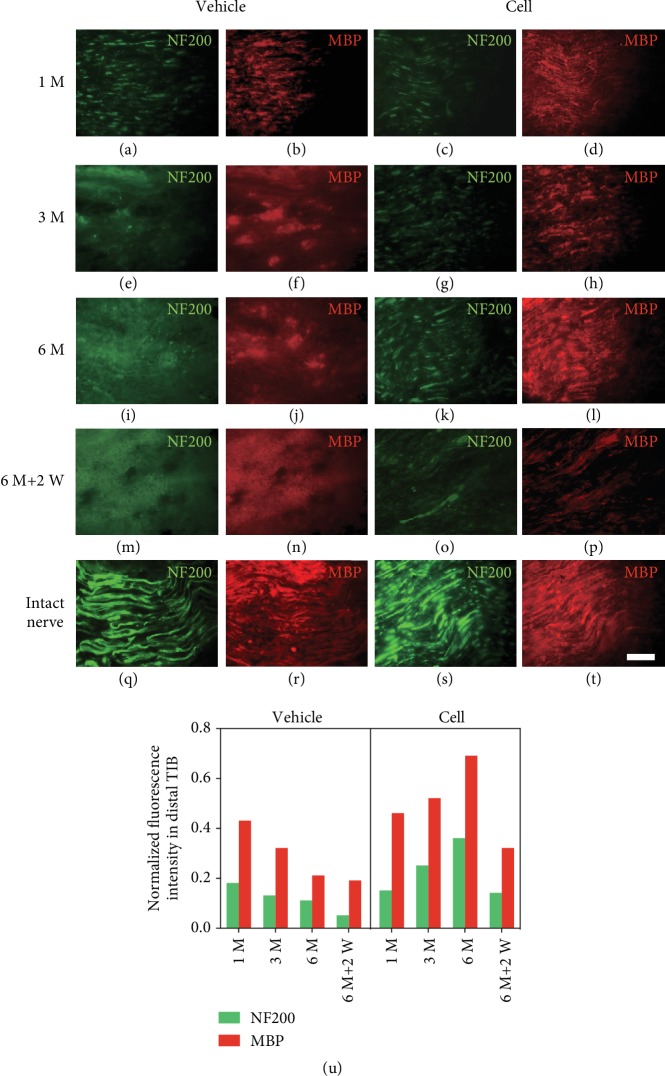
Axonal and myelin sheath status in the distal nerve stump after the 1^st^ and 2^nd^ surgery-I. Longitudinal sections of the distal part of the transplanted site were stained with NF200 (an axon marker) and MBP (a myelin sheath marker). (a–d) One month after the 1^st^ surgery, there were small amounts of myelin debris and axons in the distal stump in the vehicle group. Meanwhile, NF200-positive axons appeared in the cell group and were partially wrapped by the MBP-positive myelin sheath. (e–h) Three months and (i–l) six months after the 1^st^ surgery, the axonal and myelin debris had been gradually eliminated in the vehicle group, and abundant NF200-positive axons and MBP-positive myelin sheath were observed in the cell group. (m–p) Two weeks after the 2^nd^ surgery-I, the vehicle group showed no positive staining. Meanwhile, the distal stump in the cell group reentered a denervated status, exhibiting discontinuous axons and myelin sheath debris. (u) The normalized intensities were determined in triplicate in sections taken from 1 rat. The trend in the expression intensity of NF200-positive axons was consistent with the MBP-positive myelin sheath in both groups. A sharp decline was observed in the expression of both markers after cell excision in the cell group. Scale bar = 100 *μ*m.

**Figure 4:**
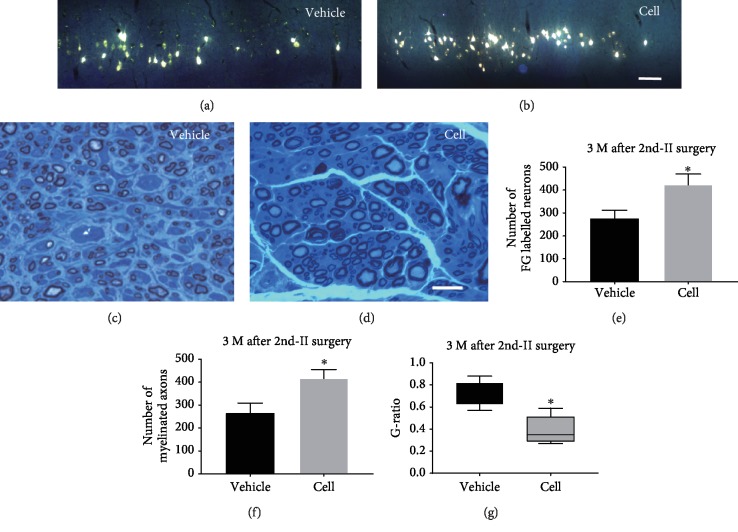
FG labeling of neurons and EM analysis of the distal TIB 3 months after the 2^nd^ surgery-II. (a, b) More FG-labeled neurons were found in the cell group than in the vehicle group. Scale bar = 100 *μ*m. (c, d) More myelinated axons were observed in the cell transplantation group than in the vehicle group. Scale bar = 25 *μ*m. (e) Statistical analysis showed significantly more regenerating neurons in the cell group than in the vehicle group (^∗^*P* < 0.05, compared with the vehicle group). (f) Statistical analysis showed significantly more myelinated axons in the cell group than in the control group (^∗^*P* < 0.05, compared with the vehicle group). (g) The G-ratio was significantly lower in the cell group than in the vehicle group (^∗^*P* < 0.05, compared with the vehicle group).

Due to the intensity of GFP attenuation in transplanted cells as mentioned above, we used NF200 to label regenerated axons and MBP to label myelin sheath. The immunoreactivity of NF200-positive axons and MBP-positive myelin sheath in the vehicle group decreased gradually as time progressed and was almost undetectable 6 months after the 1^st^ surgery, indicating long-term denervation and fibrosis status (Figures 3(a), 3(b), 3(e), 3(f), 3(i), 3(j), and 3(u)). The regeneration of NF200-positive axons coincided with the expression of MBP-positive myelin sheath, and both were gradually increased in the cell group (Figures 3(c), 3(d), 3(g), 3(h),3(k), 3(l), and 3(u)). Two weeks after the 2^nd^ surgery-I, the NF200-positive and MBP-positive signals in the cell group became discontinuous, indicating that Wallerian degeneration reemerges in axons and the myelin sheath (Figures 3(o) and 3(p)); there was no immunoreactivity in the vehicle group (Figures 3(m) and 3(n)). The fluorescent intensity of each section was normalized to the intensity of the contralateral intact CP (Figures 3(q)–3(t)). The time course of axons from transplanted cells grown into denervated CP nerve stumps in the cell group and the process of axonal gradual degeneration in the vehicle group are shown in the histograms in Figure 4(f). The expression of MBP showed the same trend as the expression of NF200.

### 3.3. The Presence and Absence of Transplanted Neurons Affected the SC Quantity and Phenotype Change in the Distal Nerve

**Figure 5:**
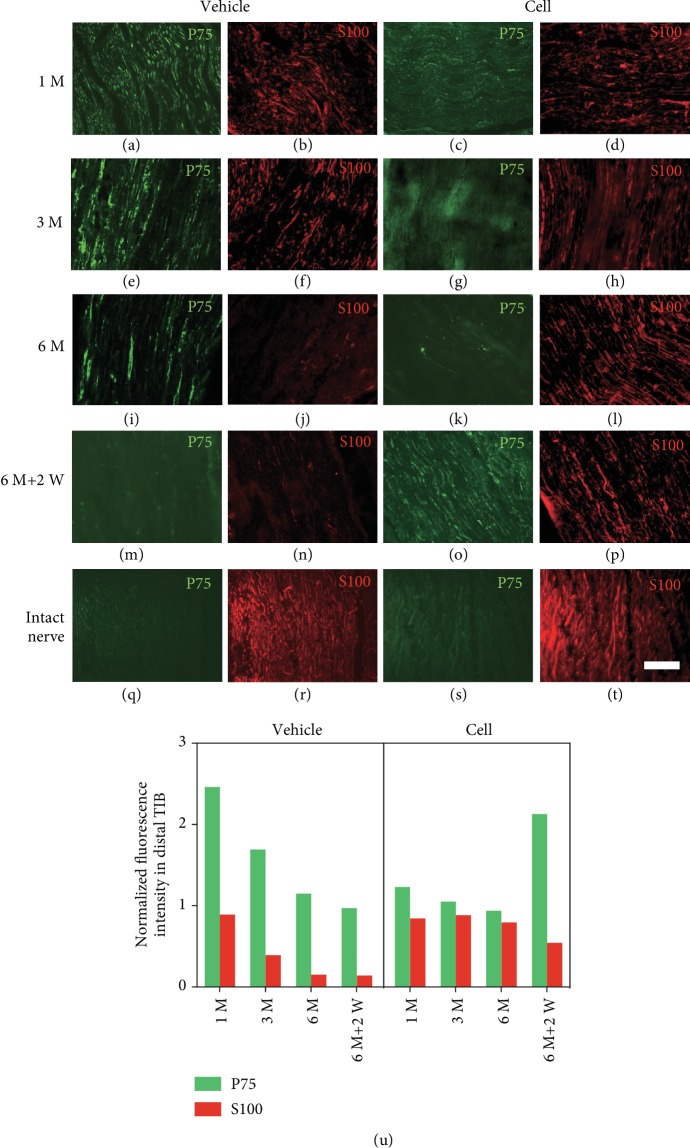
SC quantity and phenotype change in distal nerve stump after the 1^st^ and 2^nd^ surgery-I. Longitudinal sections of the distal part of the transplanted site were stained with S100 (an SC cytoplasmic marker) and P75 (a dedifferentiated SC marker). (a–d) In the vehicle group, one month after the 1^st^ surgery, the intensity of P75 was significantly higher and the intensity of S100 was lower on the transected side than on the intact side. In the cell group, the increase in P75 expression and the decrease in S100 expression were not obvious compared with the vehicle group. (e–l) At 3 and 6 months after the 1^st^ surgery, the expression of both markers had gradually decreased to a low level in the vehicle group. In the cell group, P75 was maintained at a low level while S100 expression was maintained at a high level, and their levels did not significantly fluctuate. (m–p) Two weeks after the 2^nd^ surgery-I, the expression of P75 in the cell group increased, while the expression of S100 decreased; the vehicle group did not exhibit these changes. (u) The expression intensity of P75 and S100 in the vehicle group declined over time. In the cell group, a reversal in the expression of P75 (low to high) and S100 (high to low) was observed after excision of the transplanted cells. Scale bar = 100 *μ*m.

The expression of P75 neurotrophic factor, which is a marker of dedifferentiated SCs, was significantly elevated in the vehicle group 1 month after the 1^st^ surgery (Figure 5(a)). Meanwhile, the expression of P75 in the cell group was weaker than that in the vehicle group (Figure 5(c)). As time progressed, the expression of P75 gradually decreased in both groups, and the expression of P75 in the cell group decreased faster than that in the vehicle group (Figures 5(e) and 5(g)). Six months after the first-stage surgery, the P75 immunoreactivity in the cell group returned to the baseline of myelinating SCs, while a small degree of expression remained in the vehicle group (Figures 5(i) and 5(k)). Two weeks after the second-stage surgery-I, the expression of P75 in the cell group increased again (Figure 5(o)), indicating that the SCs in the cell group underwent another process of dedifferentiation. This process was not observed in the vehicle group, which maintained a low level of expression (Figure 5(m)).

S100, the characteristic cytoplasmic marker of SCs, was used to label the quantity of SCs. One month after the 1^st^ surgery, the intensity of S100 in both groups was slightly lower than that in intact nerves (Figures 5(b) and 5(d)). Three months after the 1^st^ surgery, the intensity of S100 in the vehicle group gradually decreased, and there was only occasional visible fluorescence at 6 months after the 1^st^ surgery (Figures 5(f) and 5(j)). In the cell group, S100 was maintained at a high level at both 3 and 6 months after the 1^st^ surgery (Figures 5(h) and 5(l)). Two weeks after the 2^nd^ stage-I surgery, the expression of S100 in the vehicle group was still low (Figure 5(n)), and the expression of S100 in the cell group declined (Figure 5(p)), indicating that the number of SCs begins to decline after denervation. The fluorescent intensity of each section was normalized to the intensity of the contralateral intact CP (Figures 5(q)–5(t)).

### 3.4. The Presence and Absence of Transplanted Neurons Affected the Expression of GAGs and a Myelin-Related Gene in the Distal Nerve

**Figure 6:**
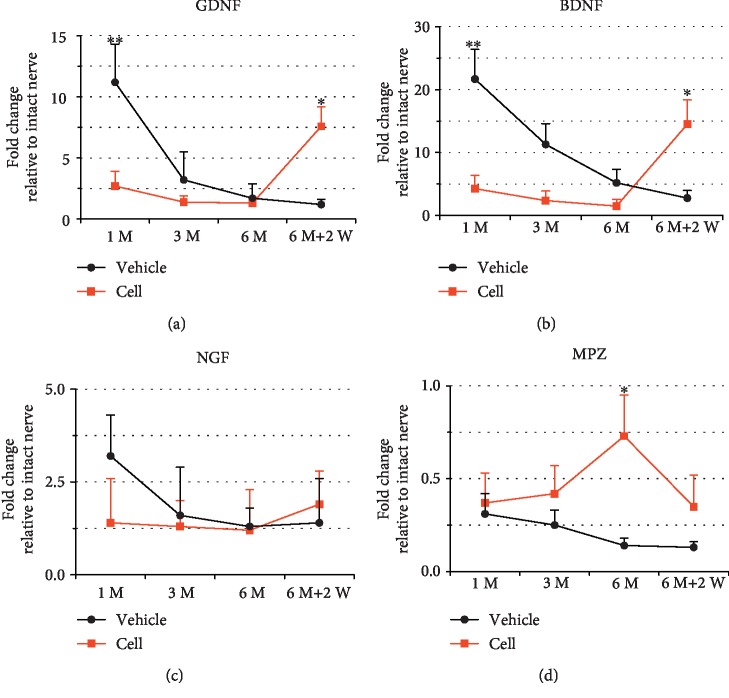
Temporal mRNA expression of growth factors in the distal nerve stump after the 1^st^ and 2^nd^ surgery-I. The transplanted neurons downregulated the expression of GAGs (a, GDNF; b, BDNF; c, NGF) and upregulated the expression of a myelin-related gene (d, MPZ). Two weeks after the 2^nd^ surgery-I, the excision of transplanted neurons upregulated the expression of GAGs and downregulated the expression of a myelin-related gene. The expression level for each gene in the distal nerve was calculated as the fold change compared with that in the contralateral intact nerve (normalized to 1 as an arbitrary unit). Asterisks placed above the standard error bars indicate a significant difference between the cell injection and vehicle injection groups at a given time point (^∗∗^*P* < 0.01 compared with the vehicle injection group, ^∗^*P* < 0.05 compared with the vehicle injection group). Error bars represent SEM.

Current evidence suggests that SCs in denervated nerves resume a more primitive, nonmyelinating phenotype, upregulate GAGs, and downregulate myelin-related genes to attract proximal axons [25]. The expression of GDNF, BDNF, NGF, and MPZ was measured by qRT-PCR at different time points, and the expression levels in the contralateral intact nerve were normalized to 1 as an arbitrary unit (Figure 6).

One month after the 1^st^ surgery, GDNF expression in the vehicle group was upregulated (by 11-fold compared with intact nerve). In the cell group, GDNF expression was also upregulated (by 3-fold compared with intact nerve) but was significantly lower than that in the vehicle injection group (Figure 6(a), ^∗∗^*P* < 0.01 compared with the vehicle injection group). Three and six months after the 1^st^ surgery, the expression levels of GDNF in the vehicle injection and cell injection groups both declined and were not significantly different (Figure 6(a), *P* > 0.05). Two weeks after the 2^nd^ surgery-I, the expression of GDNF in the cell injection group was significantly elevated, reaching 8-fold the level in the intact nerve, and was significantly higher than that in the vehicle injection group, which declined to 1.2-fold the level in the intact nerve (Figure 6(a), ^∗^*P* < 0.05 compared with the vehicle injection group). BDNF expression exhibited similar changes. One month after the 1^st^ surgery, BDNF expression was increased by 22-fold and 4-fold in the vehicle injection and cell injection groups, respectively, and the difference was significant (Figure 6(b), ^∗∗^*P* < 0.01 compared with the vehicle injection group). Three and six months after the 1^st^ surgery, the expression level of BDNF in the vehicle group declined over time; although it was still higher than the expression in the cell group, there was no significant difference between the groups (Figure 6(b), *P* > 0.05). Two weeks after the 2^nd^ surgery-I, the expression level of BDNF in the cell injection group was significantly elevated, reaching 14-fold the level in the intact nerve, and was significantly higher than that in the vehicle injection group, which declined to 2.8-fold the level in the intact nerve (Figure 6(b), ^∗^*P* < 0.05 compared with the vehicle injection group). NGF exhibited a different expression pattern from the former two factors. At one month after the 1^st^ surgery, NGF expression increased in the vehicle group (by 3.2-fold compared with the intact nerve) but gradually fell to the baseline level at a later time point. The cell group remained at a low level of NGF expression at all time points. There was no significant difference in the expression between the two groups at any time point (Figure 6(c), *P* > 0.05).

The expression of MPZ in the two groups showed the opposite trend compared with the BDNF and GDNF. The cell group showed a gradually increasing trend at 1 month, 3 months, and 6 months after the 1^st^ surgery and a decreasing trend 2 weeks after the 2^nd^ surgery. The vehicle group showed a gradual downward trend after the 1^st^ surgery. The expression of MPZ in the cell group at each time point was higher than that in the vehicle group, and the difference between the two groups at 6 months after the 1^st^ surgery was significant (Figure 6(d), ^∗^*P* < 0.05 compared with the vehicle injection group).

### 3.5. Reactivation of SCs in Grafts Promoted Axon Regeneration and Remyelination after 2nd Surgery-II

After the segment including the grafted cells was removed and a 10 mm segment of the distal stump was transplanted to bridge the freshly cut TIB nerve, the axon from the proximal TIB nerve was able to regenerate into the graft. Three months later, retrograde FG labeling and EM analysis were performed to evaluate regeneration and remyelination in the transplanted nerve. The number of labeled motor neurons in the cell group was significantly higher than that in the vehicle group (278.1 ± 34.3 vs. 423.4 ± 47.8, *P* < 0.05; Figures 4(a), 4(b), and 4(e)). Consistent with the finding of FG labeling, the number of myelinated axons in the vehicle group was more abundant in the cell group (266.2 ± 43.7 vs. 416.3 ± 49.5, *P* < 0.05; Figures 4(c), 4(d), and 4(f)). Moreover, the G-ratio (axonal diameter/total length of the myelin sheath) in the cell group was significantly higher than that in the PC group (0.71 ± 0.04 vs. 0.39 ± 0.05, *P* < 0.05; Figure 4(g)), indicating that the myelin sheath of the myelinated nerve fibers in the cell group was thicker than that in the vehicle group.

### 3.6. Reactivation of SCs in Grafts Promoted Reinnervation of the Muscle after the 2nd Surgery-II

**Figure 7:**
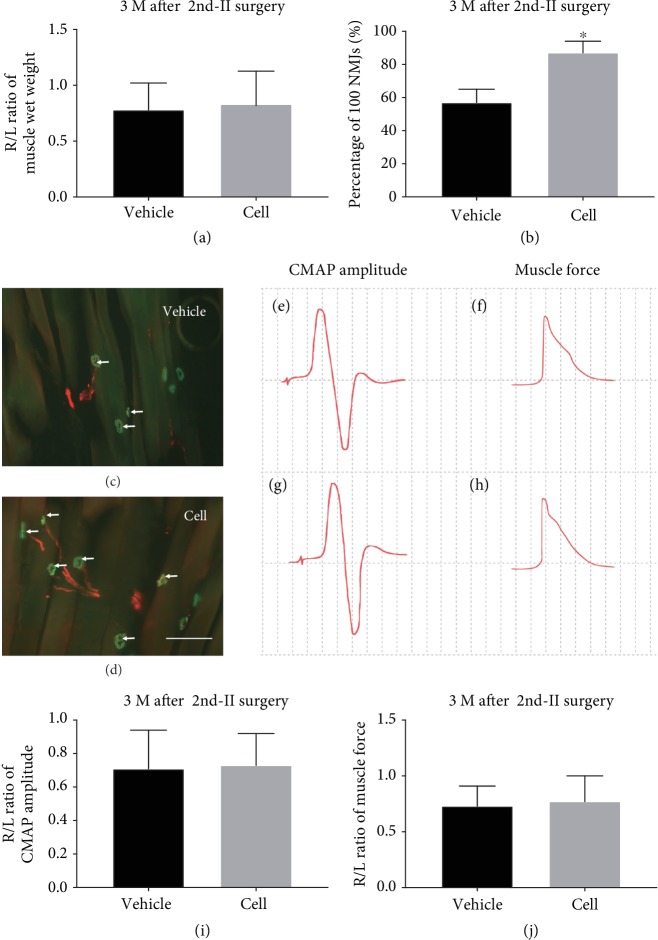
IHC and electrophysiological analysis of the GM 3 months after the 2^nd^ surgery-II. (a) There was no significant difference in the R/L wet weight ratio between the two groups (*P* > 0.05). (b–d) IHC staining of NMJs showed more reestablished NMJs (arrows) in the cell group than in the vehicle group. Statistical analysis showed a higher reinnervation rate in the cell group (^∗^*P* < 0.05, compared with the vehicle injection group). (e–h) Typical CMAP amplitude and muscle force. (i, j) The R/L ratio of CMAP amplitude and muscle force in the cell group showed no significant differences compared with the vehicle group (*P* > 0.05).

Three months after the 2^nd^ surgery-II, the muscles of each group were significantly atrophied compared with those on the contralateral side. The wet weight R/L ratios of the quadriceps muscles in the vehicle group and cell group were 0.77 ± 0.17 and 0.61 ± 0.12, respectively, which did not significantly differ (Figure 7(a), *P* > 0.05). We further analyzed the reinnervation of the denervated motor endplates by IHC staining of axon terminals and AchRs (Figures 7(c) and 7(d)). We found both denervated and reinnervated motor endplates in both groups, and the reinnervation rate was significantly higher in the cell group than in the vehicle group 3 months after the 2^nd^ surgery-II (*P* < 0.05, Figure 7(b)). Electrophysiological analysis showed that the average CMAP and muscle contractility force were slightly higher in the cell group than in the vehicle group (Figures 7(e)–7(h)), but there were no statistically significant differences (Figures 7(i) and 7(j), *P* > 0.05).

## 4. Discussion

In this study, we combined cell transplantation and nerve transfer strategies to investigate whether the transplantation of embryonic spinal cord cells could benefit the microenvironment of the distal stump of the injured nerve. A two-stage surgery model was used, and we showed that at different times after transplantation, the transplanted embryonic spinal cord cells could survive and generate neurons. Thus, the neurons play the role of proximal axons to prevent chronic degeneration and fibrosis of SCs. After excision of the transplanted cells, the SCs returned to their dedifferentiated phenotype and upregulated growth-associated gene expression. The ability of SCs to be activated again allowed a favorable microenvironment to be created and enhanced the regeneration and remyelination of proximal axons. Muscle reinnervation was also elevated. This transplantation strategy could provide a treatment option for complex neurological injuries in the clinic.

### 4.1. The Principle Underlying the Design of the Experiment

As early as 1993, Erb et al. transplanted embryonic spinal cord cells into the distal stump of an injured peripheral nerve and found that neurons could survive and regenerate axons, dominating distal muscles [26]. To promote the efficacy of transplantation, later studies introduced several methods to transform the cells by inducing differentiation, applying electrical stimulation, increasing trophic factor expression, and transfecting with light-sensitive proteins [27–30]. However, the main purpose of these studies was neuronal replacement, and it remains difficult to use the abovementioned cells to replace autologous neurons in clinical practice. Because the distal nerve stump may be less tolerating than muscle, a more practical strategy to improve regeneration is to maintain the distal nerve in an “available” state to accept the proximal axons [17]. A previous study used mesenchymal stem cell transplantation to activate denervated SCs in predegenerated nerves, but because the denervation time of the distal stump is short, the true utility of this approach is uncertain [31]. The current “side-to-side,” “side-to-end,” “end-to-side,” and “supercharge end-to-side” surgical methods in clinical practice are used to “babysit” distal nerves while waiting for the arrival of proximal regenerating axons [10, 32, 33]. Inspired by the clinical “babysit” concept, after transplanting cells to protect the distal nerve for a period of time, we cut off the cells and terminated the protection. The role of transplanted cells is “mobile power charging” without relying on surrounding donor nerves. The purpose of this study is to demonstrate that the presence and absence of transplanted cells have a regulatory effect on SCs in the distal nerve and this regulation can improve the ability of SCs to induce proximal axons.

In our previous studies, it was also proven that the reexcision of transplanted cells promoted the regeneration of axons after secondary replantation, although the degeneration time of the distal nerves in the experimental model was only 3 months [13]. Gordon et al. found that the proliferation and phenotypic changes of SCs in the distal nerves after peripheral nerve transection were maintained until one month after surgery. With prolonged denervation time, the number of SCs and the expression of GAGs in the distal nerves gradually decrease. Six months after transection, SCs are significantly atrophied, and the reversion of growth factor expression to baseline levels makes axonal regeneration difficult to induce [15]. Therefore, the time of chronic neurodegeneration was extended to 6 months in this study. Moreover, our previous research used a cross-suture model, which was unable to distinguish cells from promoting regeneration through the action of distal nerves or muscles. The mechanism underlying the phenomenon was not clearly explained. This study used a cell-treated graft to bridge fresh nerve axotomy, thus excluding the effects of muscle factors on the results, demonstrating that cell-treated grafts do improve the regeneration of proximal axons. Changes in the SC phenotype and GAG expression were also detected at different time points after cell transplantation and after excision of the transplanted cells to explore the interaction between transplanted cells and peripheral nerve SCs.

### 4.2. Innervation by Transplanted Neurons Maintained the Quantity and Myelinated Phenotype of SCs

During peripheral nerve regeneration, SC proliferation was enhanced by growth-associated proteins (GAPs), such as neuregulin and GAP-43, which are secreted by proximal axons. When SCs do not receive a proximal signal for a long period of time, they gradually degenerate and decrease in number [34]. In the present study, during the 6-month period of survival of transplanted cells, S100 IHC staining suggested that the number of SCs in the distal nerves was maintained at a high level, while the SCs in the vehicle group gradually degenerated, exhibited fibrosis, and decreased in number. Thus, transplanted cells may replace proximal axons and play a role in maintaining the number of SCs, laying the foundation for promoting regeneration after the 2^nd^ surgery.

In addition to proliferation, dedifferentiated SCs transform the myelin-formation phenotype into a growth-supportive phenotype. The expression of myelin-related genes decreased, and the expression of dedifferentiated SC marker P75 and GAGs such as GDNF, BDNF, and NGF was upregulated [35]. Consistent with previous research, one month after the 1^st^ surgery, P75 and the trophic factors GDNF and BDNF were highly expressed in the vehicle group. It was noted from our study that NGF was not statistically different at all time points. This may be due to the fact that NGF is a traditional sensory neurotrophins, the expression of which will be upregulated in denervated sensory nerve (cutaneous-derived SCs), but its degree of upregulation was significantly weaker than that of another sensory neurotrophins “BDNF.” Moreover, the CP was a mixed nerve and predominated was motor axons, after denervation, the motor-derived SCs may not upregulate the expression of NGF. Together, these two factors lead to the upregulation of NGF in the vehicle group not as significant as BDNF and GDNF in the denervated CP. This is consistent with a previous report that the expression of NGF was not significantly different in the denervated nerve and immediately repaired nerve [14].

When proximal axonal growth occurs, SCs gradually change from a dedifferentiated phenotype to a mature phenotype when they form myelin to surround axons to support nerve impulse conduction, and therefore, the expression of P75 and GAGs declines [14, 35, 36]. Based on the IHC results, we confirmed that after the 1^st^ surgery, in the cell group, neurons gradually emitted axons to innervate the distal nerves, while SCs gradually formed myelin sheaths to surround axons over time. The distal nerve switched from a denervated state to an innervated state. The expression of P75 was significantly lower in the cell group than in the vehicle group at 1 month after the 1^st^ surgery, suggesting a decrease in dedifferentiated SCs. Similarly, the expression of regeneration-related factors was also significantly lower in the cell group than in the vehicle group. When more SCs were converted to mature phenotypes, the expression of P75 and regeneration-related factors in the cell group decreased slowly. SCs in the vehicle group decreased their P75 and trophic factor expression levels at a faster rate over time, which were similar to the levels in the cell group at 6 months after the 1^st^ surgery. The growth-supportive phenotype cannot be maintained for a long period of time without proximal axons, and thus, SCs eventually enter a chronic degenerative and fibrotic state [14, 34, 37].

### 4.3. Reactivation of SCs by Transplanted Neuron Excision Promoted Axon Regeneration and Remyelination

Six months after the 1^st^ surgery, minimal positive staining for P75 was found in the distal nerve in both groups. The expression of GAGs in the two groups was also at a low level close to baseline. However, the expression of the myelin gene was still active distal to the cell transplanted site in the cell group, indicating that remyelination was ongoing. MBP staining also confirmed gradual formation of the myelin sheath over time in the cell group. These phenomena were absent in the vehicle group.

Two weeks after the 2^nd^ surgery-I, the expression of P75 in the distal cells of the cell group again increased. The levels of neurotrophic factors GDNF and BDNF were significantly higher than the baseline levels before excision, while the expression of the myelin gene was significantly lower than before excision. The vehicle group did not exhibit this effect, suggesting that resecting the cell transplanted site is equivalent to cutting off the proximal axon again. The SCs in the distal stump of the cell transplantation site undergo a “fresh degeneration” process and convert to a dedifferentiated phenotype. The function of SCs shifts from forming myelin to guiding axon regeneration. In the vehicle group, SCs undergo long-term degeneration for 6 months and become atrophic and fibrotic, and even if the proximal nerve segment is removed, a state of dedifferentiation cannot be reinitiated. These results also indirectly suggest an interaction between the transplanted cells and the distal segment of SCs, and the effect provides an ideal environment for axonal regeneration.

In the 2^nd^ surgery-II, the redegeneration distal CP nerve was used as a graft to bridge the freshly cut TIB nerve end. The results also demonstrated that the cell-protected nerve segment promoted the regeneration of proximal axons, which verified our hypothesis. The EM analysis also revealed that the remyelination of regenerated axons was significantly improved, indicating that the SCs in the graft did not lose their ability to form myelin after redegeneration, but rather, this ability was enhanced. Furthermore, we examined the recovery of muscle after the 2^nd^ surgery-II and found that the reinnervation rate of the GM in the cell group was increased, although there were no significant differences in muscle atrophy and EMG function. In a study by Gordon et al., the degree of muscle atrophy and contraction force after bridging surgery was similar between the chronic denervation graft group and the fresh graft group, which is consistent with our results [4]. A possible explanation is that although the number of axons through the chronic denervation graft is insufficient, the limited number of formed motor units can compensate for the loss of quantity by the increased function of a single motor unit, thereby achieving functional recovery and alleviating atrophy. In our previous study, the model protected both the distal nerves and muscles, and a difference in muscle atrophy and function was found after the second-stage repair. The findings proved that transplanted cells have a separate protective effect on muscles, although the mechanism requires further study [13]. These data prove our original argument that the activation of chronic SC denervation but not chronic denervation of muscles by our strategy improves axon regeneration and reinnervation independently.

There are several limitations of this study. First, our study lacked a noninjected control group containing animals that did not receive an injection. It is possible that the second surgery itself may have an effect on the distal stump even without the implantation of cells. Furthermore, the embryonic spinal cord cells were a mixture of neurons and neural progenitor cells [38]. We lacked a group of other cells (such as glial cells) and cannot rule out the possible effects of factors secreted by glial cells on the results; a more pure neuron source, such as induced pluripotent cells, would be ideal. Moreover, the mechanisms underlying the protection of the denervated muscle from atrophy remain to be elucidated, and further studies from a molecular perspective are required.

## 5. Conclusions

Neurons transplanted into the distal stump of the injured nerve can interact with SCs, playing the role to some extent of proximal axons and preventing chronic degeneration and fibrosis of SCs. The ability of SCs to be activated again after resection of the neurons allows a favorable microenvironment to be created for promoting the growth of proximal axons. This transplant strategy could provide a treatment option for complex neurological injuries in the clinic.

## Data Availability

The data used to support the findings of this study are included within the article.
